# “Be kind to young people so they feel at home”: a qualitative study of adolescents’ and service providers’ perceptions of youth-friendly sexual and reproductive health services in Vanuatu

**DOI:** 10.1186/1472-6963-13-455

**Published:** 2013-10-31

**Authors:** Elissa C Kennedy, Siula Bulu, Jennifer Harris, David Humphreys, Jayline Malverus, Natalie J Gray

**Affiliations:** 1 Centre for International Health, Burnet Institute, 85 Commercial Rd, Melbourne, Victoria, Australia; 2 School of Public Health and Preventive Medicine, Monash University, Victoria, Australia; 3 Wan Smolbag Theatre, Port Vila, Vanuatu

**Keywords:** Adolescent, Adolescent health services/utilization, Adolescent health services/standards, Reproductive health services/utilization, Health services accessibility, Focus groups, Pacific, Vanuatu

## Abstract

**Background:**

Sexual activity during adolescence is common in Vanuatu, however many adolescents lack access to sexual and reproductive health (SRH) services and subsequently suffer a disproportionate burden of poor SRH. There is limited peer-reviewed research describing adolescents’ SRH service delivery preferences in Vanuatu to inform policy and programs. The aim of this qualitative study was to explore the barriers preventing adolescents from accessing SRH services in Vanuatu and the features of a youth-friendly health service as defined by adolescents.

**Methods:**

Sixty-six focus group discussions were conducted with 341 male and female adolescents aged 15–19 years in rural and urban communities. Additionally, 12 semi-structured interviews were undertaken with policymakers and service providers. Data were analysed using thematic analysis.

**Results:**

Socio-cultural norms and taboos regarding adolescent sexual behaviour were the most significant factors preventing adolescents from accessing services. These contributed to adolescents’ own fear and shame, judgmental attitudes of service providers, and disapproval from parents and community gate-keepers. Lack of confidentiality and privacy, costs, and adolescents’ lack of SRH knowledge were also important barriers. Adolescents and service providers identified opportunities to make existing services more youth-friendly. The most important feature of a youth-friendly health service described by adolescents was a friendly service provider. Free or affordable services, reliable commodity supply, confidentiality and privacy were also key features. The need to address socio-cultural norms and community knowledge and attitudes was also highlighted.

**Conclusions:**

There are significant demand and supply-side barriers contributing to low utilisation of SRH services by adolescents in Vanuatu. However, there are many opportunities to make existing SRH services more youth-friendly, such as improving service provider training. Investment is also required in strategies that aim to create a more supportive environment for adolescent SRH.

## Background

Adolescents aged between 10 and 19 years account for 20% of the population of Pacific Island countries [1]. Sexual activity is common during adolescence: up to 65% of girls and 72% of boys aged 15–19 in this region have ever had sex. Early sexual debut (less than 15 years) is also common, with as many as 15% of girls and 35% of boys reporting sex before the age of 15 [2]. Many are ill-prepared for this transition, lacking comprehensive knowledge about sexual and reproductive health (SRH) and facing significant barriers to accessing quality SRH services [3]. Consequently adolescents in the Pacific suffer a disproportionate burden of poor SRH, including high rates of sexually transmitted infections (STIs) and unintended pregnancy, often with significant socio-economic consequences [2,4].

Vanuatu is a Melanesian country and one of the poorest in the Pacific [5]. Limited data indicate that around 10% of young people in Vanuatu have had sex by the age of 15, with the median age of sexual debut 16.7 years for males and 17 years for females [6,7]. Less than 15% of sexually active youth (aged 15–24) report consistent condom use and fewer than 35% of adolescent girls aged 15–19 who are married or in union use a modern method of contraception [7,8]. More than 36% of ni-Vanuatu males and 14% of females aged 15–24 report ever being diagnosed with an STI, with surveillance data indicating the highest rates of chlamydia occur among adolescents aged 15–19 [7,9]. Adolescent fertility is relatively high (66 births per 1000 girls aged 15–19), and adolescent girls account for almost one in eight births [10,11].

The majority of SRH services in Vanuatu are provided by government facilities, with a small number of youth-oriented clinics provided by non-government organisations. Traditional healers are an important part of the informal health system in Vanuatu, particularly in more remote islands, although less is known about their use by adolescents [12,13]. Low utilisation of mainstream services is a significant barrier to improving adolescent SRH, a challenge noted by the Ministry of Health [14,15]. While global guidance regarding youth-friendly health services exists [16,17], there is limited peer-reviewed research describing barriers and service-delivery preferences of adolescents in Vanuatu to guide policy and programs. The need for qualitative information about adolescents’ perceptions of SRH services has also been noted by UNFPA following a situational analysis of adolescent SRH in Vanuatu [18].

The primary aim of this qualitative study was to explore the barriers, enablers and SRH information and service delivery preferences of adolescents aged 15–19 years in Vanuatu. The secondary aim was to explore attitudes and perceptions of service providers and policymakers regarding the provision of SRH information and services to adolescents. This paper focuses on the barriers to accessing SRH services and describes the features of a youth-friendly health service as defined by adolescents.

## Methods

### Study setting

The Republic of Vanuatu is an archipelago nation of more than 80 islands spread across 612,300 square kilometres of the South Pacific. The population of 239,000 is predominantly rural, with around a quarter living in the urban centres of Port Vila and Luganville [10]. This study was conducted on the two most populous islands, Efate and Espiritu Santo, home to 48% of the country’s population of 15–19 year olds [10]. These islands include the only two urban centres in Vanuatu, in addition to rural and remote communities, so were selected for this study to enable exploration of the perspectives of both urban and rural adolescents. Additionally, the local research partner was based in Efate and Espiritu Santo, enabling greater access to, and engagement with, communities on these islands.

### Study design

The study design was developed following a review of the available data describing adolescents’ access to SRH interventions and outcomes in the Pacific, a review of global peer-reviewed literature describing barriers and enablers to accessing SRH information and services (including the available peer-reviewed literature from the Pacific), and following consultation with Vanuatu stakeholders, including the Ministry of Health.

Qualitative methods were used to investigate adolescents’, service-providers’ and policymakers’ needs, attitudes, perceptions and experiences related to SRH services. Focus group discussions (FGDs) were conducted with adolescents to explore three broad areas: current sources and perceptions of SRH services; barriers to accessing services; and, SRH service delivery preferences. In this study SRH included: STIs, including HIV; family planning (FP); post-abortion care (as abortion is highly legally restricted); pregnancy testing; and, pregnancy care. FGDs were used to capture a wide range of views and enable interaction between participants with differing experiences accessing services, stimulating discussion and providing greater insight into attitudes and perceptions. Additionally, 12 interviews were conducted with policymakers and health service providers (government and non-government) to explore attitudes towards adolescent SRH, and identify supply-side barriers and opportunities to strengthen the provision of youth-friendly health services.

### Sampling and recruitment of participants

Adolescent participants were recruited from 27 communities, including 15 urban and 12 rural communities. Communities were identified by the local research partner and youth peer educators and included communities in which they had current health promotion activities, as well as those with which they had no prior engagement. Nine schools (five urban, four rural) were also invited to participate. These included the main urban secondary schools as well as boarding schools with large numbers of students from rural communities. Schools were selected by the local research partner and included those where they had previously provided health promotion activities and had an existing relationship.

Before recruitment, meetings were held between the research team and community leaders, parents, youth leaders and school staff to provide information about the study purpose and design. Written consent was obtained from each community leader, youth leader or school principal, providing permission for their community to participate in the study. No communities refused to participate.

In and out-of-school adolescent males and females were eligible to participate if they were aged between 15–19 years. After the initial meetings, youth leaders, peer educators, school staff and community leaders advertised the time and day of each FGD. All eligible adolescents attending school or youth centres, or present in the community on that day, were invited by youth peer educators to participate in the study. Additionally, four FGDs were conducted with females aged 15–19 who had ever been pregnant and four with males aged 15–24 who had ever fathered a child to examine perceptions of those who were likely to have had contact with SRH services. These adolescents were identified by peer educators familiar with the participating communities.

Policymakers and providers were identified following mapping of health services on both islands and were recruited by the research team. Policymakers included representatives from the Ministry of Health and multilateral agencies. Service providers were recruited from the two main government hospitals on both islands, government clinics, non-government primary level service providers and one school nurse. The names of included organisations/health facilities are not listed to preserve confidentiality.

### Data collection

FGDs and interviews were conducted over a six-month period in 2010. An open-ended FGD question guide was developed by the research team based on a review of existing literature and in consultation with the local research partner and the Vanuatu Ministry of Health. A structured vignette about two fictional adolescents was used to contextualise the discussion and provide a less personal and threatening means of exploring potentially sensitive issues. This technique uses a fictional story or scenario to explore actions, judgments and cultural norms related to a specific situation or context. While there is limited literature describing their use in FGDs, some authors have noted that a vignette can help stimulate discussion and may be particularly useful when discussing sensitive topics [19-21]. The short story used in this study depicted a new relationship between two adolescents that unfolded over stages, with participants asked to respond to occurrences or dilemmas at different stages. The vignette was developed with youth peer educators, drawing on experiences of young people to ensure the story was plausible and culturally appropriate. A participatory activity (rank ordering) that enabled participants to rank the features of a youth-friendly health service was conducted with half the FGDs. The list of features presented to participants was developed following a review of the literature and discussion with youth peer educators. In addition, all FGD participants completed an anonymous questionnaire concerning basic demographic information. The questionnaire was verbally administered to the group by a facilitator and collected before each FGD. No identifying details were recorded on the questionnaire. Data collection tools were pre-tested with youth peer educators.

To observe cultural sensitivity and encourage open discussion, male and female FGDs were conducted separately and moderated by a facilitator of the same sex. FGDs were also divided by age group: 15–17 year olds and 18–19 year olds. Due to the multiple data collection sites (urban and rural communities across two islands) and the division of groups according to age and sex, multiple male and female teams were trained to conduct the FGDs. For logistical reasons, data collection by the different teams largely occurred simultaneously, which limited the opportunity to analyse data during collection. For this reason, it was not possible to accurately assess data for saturation prior to analysing the translated transcripts. Therefore, a large number of FGDs were planned to attempt to capture the breadth of opinions and experience across the different groups.

FGDs comprising up to eight participants and lasting 60–90 minutes were conducted in Bislama by ni-Vanuatu youth peer educators. Three FGDs included only two participants, and were included in the analysis. Peer educators selected to facilitate FGDs were aged over 15 years, had previously been trained by Wan Smolbag Theatre, and were experienced in discussing sensitive issues related to SRH with adolescents. They received additional training from the research team in qualitative research methods, FGD facilitation and research ethics. One to two trained note-takers recorded hand-written notes during each discussion. These were translated into English by a bilingual researcher and then reviewed by note-takers and facilitators during workshops to ensure accuracy and check translation. Key informant interviews were conducted with four policymakers and eight service providers in English using a semi-structured question guide and recorded by a trained note-taker.

### Data analysis

Transcripts were thematically analysed using an inductive approach [22]. Three researchers read and re-read transcripts to become familiar with the data. Transcripts were annotated with initial codes relevant to the research questions which formed the initial coding frame, and broadly related to: current use and perceptions of SRH services; barriers to accessing services; enabling factors; and, features of a youth friendly health service. The three researchers independently coded transcripts and met regularly to review for consistency. Discrepancies were resolved through discussion and/or input from the local research partner New codes were added as they emerged and analysis continued until no new codes were identified. Matrices were created summarising the coded data to determine the frequencies of codes. Similar codes were then grouped into themes and sub-themes and reviewed to identify meanings and relationships between themes. Quotes were recorded to illustrate themes. Findings were validated with the local research partner, including the research officer responsible for translating all transcripts, and through two data analysis workshops held with facilitators and peer educators. Analysis of the rank ordering activity was done by allocating a score to each youth-friendly health service feature based on the order it was ranked. Scores were tallied to identify the overall and sex-disaggregated order of features from most to least important. The discussion accompanying the activity was recorded by note takers and analysed with the FGD transcripts. Quantitative data from the questionnaires administered to all FGD participants were analysed using Microsoft Excel (Microsoft Corp, Redmond, WA, USA).

### Ethical considerations

Ethics approval was granted by the Alfred Hospital Ethics Committee (Australia) and the Vanuatu Ministry of Health Research and Ethics Committee. In addition to written community consent, written consent was obtained from all individual participants. Verbal consent, witnessed by a peer educator, was obtained from those participants with limited literacy. No personal identifying information was recorded on questionnaires or transcripts, and confidentiality was explained and agreed to by all participants prior to the commencement of each FGD. At the conclusion of each FGD adolescent participants received printed health promotion materials and details of available youth friendly health services.

## Results

### Characteristics of adolescent focus group participants

A total of 66 FGDs were conducted involving 341 participants, 49.0% of whom were from rural areas (Table 1). Twenty FGDs were conducted in schools. The median age of participants was 17 years. Half were currently in school at the time of data collection and of those who had completed education, 47.3% had attended secondary school. The majority of participants were never married. Around half (50.4%) reported ever having sex, with more male participants reporting having had sex than females (59.2% versus 41.9%). Of those who were sexually experienced, the majority had commenced sexual activity between the ages of 15–19, although 8.5% reported sexual debut before the age of 15. Around three quarters of participants had heard of STIs and FP. Forty-three participants had accessed SRH services in the previous 12 months (16% of females and 9% of males).

**Table 1:** Background characteristics of adolescent focus group participants

**Background characteristics**	**Female *(N = 172) n (%)***	**Male *(N = 169) n (%)***	**Total *(N = 341) n (%)***
*Age (years)*			
15	39 (22.7)	39 (23.1)	78 (22.9)
16	20 (11.6)	28 (16.6)	48 (14.1)
17	30 (17.4)	26 (15.4)	56 (16.4)
18	40 (23.3)	27 (16.0)	67 (19.6)
19	43 (25.0)	32 (18.9)	75 (22.0)
20-24*	-	15 (8.9)	15 (4.4)
Unknown	0 (0.0)	2 (1.2)	2 (0.6)
*Location*			
Urban	96 (55.8)	78 (46.2)	174 (51.0)
Rural	76 (44.2)	91 (53.8)	167 (49.0)
*Education*			
Currently in school	93 (54.1)	85 (50.3)	178 (52.3)
Completed school	69 (40.1)	79 (46.7)	148 (43.4)
Highest level attended^‡^			
Primary	39 (56.5)	39 (49.4)	78 (52.7)
Secondary	30 (43.5)	40 (50.6)	70 (47.3)
Never attended school	2 (1.2)	3 (1.8)	5 (1.5)
No response	8 (4.7)	2 (1.2)	10 (2.9)
*Marital status*			
Never married	152 (88.4)	156 (92.3)	308 (90.3)
Ever married	5 (2.9)	5 (3.0)	10 (2.9)
No response	15 (8.7)	8 (4.7)	23 (6.7)
*Sexual activity*			
Never had sex	100 (58.1)	69 (40.8)	169 (49.6)
Ever had sex	72 (41.9)	100 (59.2)	172 (50.4)
Sexual debut			
<15	11 (6.4)	18 (10.7)	29 (8.5)
15-16	22 (12.8)	36 (21.3)	58 (17.0)
17-19	29 (16.9)	27 (16.0)	56 (16.4)
20-24	-	2 (1.2)	2 (0.6)
Unknown	2 (1.2)	11 (6.5)	13 (3.8)
No response	8 (4.7)	6 (3.6)	14 (4.1)
*Ever heard of STIs*			
No	36 (20.9)	47 (27.8)	83 (24.3)
Yes	129 (75.0)	120 (71.0)	249 (73.0)
No response	7 (4.1)	2 (1.2)	9 (2.6)
*Ever heard of family planning*			
No	42 (24.4)	44 (26.0)	86 (25.2)
Yes	124 (72.1)	121 (71.6)	245 (71.8)
No response	6 (3.5)	4 (2.4)	10 (2.9)
*Accessed SRH services in the last 12 months*			
No	118 (68.6)	122 (72.2)	240 (70.4)
Yes	28 (16.3)	15 (8.9)	43 (12.6)
Unsure / no response	26 (15.1)	32 (18.9)	58 (17.0)

*A total of 4 FGDs were carried out with young men aged 15–24 who had fathered a child.

^‡^Primary school includes Years 1–8, secondary school Years 9–13.

### Adolescents’ health seeking behaviour

The most commonly reported reason for accessing SRH services was to seek information or advice. While some groups described accessing testing or care for STIs and pregnancy, the main value of SRH services was in prevention of illness or unwanted pregnancy and was described in general terms of protecting their health and future. All groups agreed that adolescents would seek services from clinics or hospitals, although key informants noted low utilisation of mainstream services by adolescents, particularly boys, and some adolescents reported they would only attend if they were 'very sick’. Non-government organisations were highlighted as important providers of SRH services for adolescents. They were reported by adolescents and key informants to be more accessible than government facilities, most notably because non-government service providers were perceived to be more friendly and competent in dealing with adolescents:

*“KPH [NGO clinic] is better because they explain things well and talk kindly to clients.”* (Female 18–19 years, FGD)

Traditional healers were identified as providers of SRH services in half the groups, with some suggesting that they were more affordable than clinics. Some adolescents described accessing traditional healers for specific problems such as STI or abortion:

*“If Jimmy [fictional character] wants the girl to have an abortion they must go to a traditional healer.”* (Female 18–19 years, FGD)

### Barriers to SRH services

Adolescents identified a number of demand and supply-side barriers affecting access to SRH services (Table 2).

**Table 2:** Barriers to accessing sexual and reproductive health services reported by adolescents

**Demand-side barriers**	**Supply-side barriers**
Socio-cultural norms and taboos regarding sex and adolescent sexual behavior:	Judgmental attitudes of health service providers
- Stigma and shame	Cost of services and commodities
- Fear of disclosure	Lack of privacy and confidentiality
- Fear of being seen attending services	Lack of services/skilled service providers
- Opposition and disapproval from parents and communities	Inconvenient location of services
- Community or religious 'rules’ that inhibit discussion of sex or access to services	Insufficient time for counselling
Uncertainty about what they will be asked by a service provider and/or anxiety about physical examination	Unreliable commodity supply
Lack of knowledge about SRH and services	
Lack of experience attending health services	

#### ***Socio-cultural norms and taboos***

Fear and shame related to socio-cultural norms and attitudes regarding adolescent sexual behaviour were the most significant reasons why adolescents found it difficult to access SRH services. This contributed to a perception among adolescents that they were 'underage’ or 'too young’ to be sexually active or seek SRH services and fear of disclosing sexual activity to judgmental providers.

*“They are ashamed to go [to clinic] because they are underage, afraid they will get scolded.”* (Female 18–19 years, FGD)

Negative attitudes of parents and community leaders contributed to adolescents’ fear. Many described parents as a barrier, particularly for girls, either because parents directly prevented them from accessing services or because they were afraid of the consequences if their parents found out. Opposition of religious leaders to adolescent sexual activity and provision of information and services was noted as a barrier by some adolescents. *Kastom* was also an important barrier. *Kastom* refers to traditional culture, knowledge and customs and defines values, social and cultural practices of everyday life. The role of *kastom* was often described in terms of 'community rules’, which were upheld by community leaders (chiefs), contributed to stigma around adolescent sexual behaviour, inhibited open discussion of sexual matters, and prevented services (such as condom distribution) being provided in some communities:

*“[Young people are] not always free [to access SRH services] because of community rules or parents stop us.”* (Female 15–17 years, FGD)

Cultural taboos preventing discussion of sex and reproduction also contributed to adolescents’ reluctance to discuss SRH with providers, anxiety about being asked sensitive questions and also fear of physical examination. Providers also described religion and *kastom* as factors that prevented them from providing SRH advice or services to adolescents or made them uncomfortable discussing sex:

*“….the important reproductive health issues I don’t talk about because I am not allowed to talk about condoms. I don’t feel good. We have many problems but we don’t talk about them…..some communities and churches you can’t.”* (Nurse, interview)

This was particularly the case in rural areas where limited services meant that the only available provider might be an adolescent’s relative:

*“If they know the nurse, like maybe an aunty or uncle, they won’t come. Kastom is that you cannot. They are frightened with uncles and aunties because it is taboo to talk about that [sexual health] with your relatives.”* (Nurse, interview)

#### ***Judgmental attitudes and lack of skills of service providers***

Nearly all groups described a fear of unfriendly and judgmental providers, most concerned that they would be 'lectured’, 'scolded’ or made to feel ashamed for being sexually active, or experiencing an unintended pregnancy or STI:

*“Sometimes they [nurses] talk strongly to young people and tell them “it’s good you are getting this [STI] because you sleep around too much”.”* (Female 18–19 years, FGD)

Adolescents also suggested that some judgmental providers who disapproved of adolescent sexual behaviour would deny them services:

*“Young people ask for condoms but health workers don’t want to give them.”* (Male 18–19 years, FGD)

Providers themselves acknowledged that their own attitudes were one of the main reasons that adolescents did not access services:

*“Because if you are a young boy or young girl and you go there asking for family planning or condoms and the nurse might say “you are a young girl or young boy so you don’t need to use that”. Like if I was a nurse in community and see young people coming, I will not agree for young people to be practicing sex at a very early age. Because some of our nurses in our communities they will not allow and they will talk. If the nurse’s attitude is different to what young people are thinking then it’s a barrier.”* (Nurse, interview)

Some adolescents and key informants reported that providers lacked skills (particularly counselling skills) or gave poor quality care to adolescents. Many described concerns about being 'rushed’ by nurses or being given the wrong advice or treatment by poorly trained providers:

*“When they [nurses] talk about sex they don’t know what they’re talking about.”* (Male 15–17 years, FGD)

#### ***Lack of confidentiality and privacy***

Many adolescents and providers described adolescents’ fear of others finding out they had attended SRH services. In particular they were afraid of their parents, of being teased or talked about by friends, and being the victim of community 'gossip’. Some were also concerned that their partner would think that they had an STI or had been unfaithful if they knew they had attended SRH services. The lack of privacy at hospitals and government clinics was emphasised, resulting in fear of being seen by friends, relatives or community members:

*“They are afraid of gossip, that it won’t be confidential, that their partner will find out they have other partners.”* (Female 15–17 years, FGD)

*“You are worried that people will talk about you. Some are afraid that lots of people will see them.”* (Female 18–19 years, FGD)

*“You know we have government facilities but the young people they don’t feel comfortable because people will talk or maybe people will say 'I saw this young girl in clinic and maybe she has STI’.”* (Nurse, interview)

In addition, adolescents and key informants described a lack of trust in providers and concern that confidentiality would not be maintained, particularly in small communities where providers were likely to be known to the adolescent and their family:

*“They are frightened that maybe I will tell everyone that they have an STI. They want to be sure that everything will be secret. If they are not sure about the confidentiality they will be frightened.”* (Nurse, interview)

*“Some of our nurses, when a young person goes and gets condoms or family planning, some of them would actually tell the parents. But that’s not good medical practice, but it does happen.”* (Policymaker, interview)

#### ***Cost and availability of services***

Most groups agreed that the costs of services, commodities and transport were barriers for many adolescents due to high unemployment and little access to household resources. Some reported that they would be too embarrassed to ask their parents for money to attend SRH services. Almost all groups said that having to pay for SRH services and commodities would prevent them from seeking care, although some adolescents reported that if it was important they would find the money:

*“If you have to go I will still pay because it is a matter of my health.”* (Female 18–19 years, FGD)

The lack of services and distance to care were noted by some adolescents, particularly in rural areas. Not being able to see a provider of the same sex was a barrier, particularly for girls:

*“If a woman goes and there is a male nurse she will be afraid to tell him about her problems.”* (Female 15–17 years, FGD)

Unreliable commodity supply (of medicines and contraceptives) was also a noted barrier:

*“Sometimes they [nurses] don’t want to give you medicine because then they won’t have enough.”* (Male 15–17 years, FGD)

#### ***Lack of knowledge and experience***

Adolescents described a lack of 'awareness’ about SRH as a reason why they didn’t access services. There was a perception that services were only for married people or mothers and not available to adolescents. Inadequate knowledge about condoms and contraception was the major reason for not using family planning. Lack of knowledge about what they would be asked or what would happen at the clinic and not knowing how to talk with nurses were also reasons for not accessing services. A lack of experience attending a health service contributed to anxiety, as did misinformation or discouragement from friends:

*“Friends could stop you or you hear a bad story for example, sometimes when you use female condoms it can get stuck inside a woman’s vagina.”* (Male 18–19 years, FGD)

### Increasing adolescents’ access to SRH services

#### ***Creating a supportive environment for adolescent SRH***

Adolescents described the need for more 'awareness’ provided in communities and schools to increase knowledge about SRH and available services. Many reported that it was easier for adolescents who were well informed to make the decision to seek care:

*“Chiefs should allow awareness in the communities so people can hear about services. Health workers must talk with young people about the clinic services.”* (Male 18–19 years, FGD)

*“It’s easy for young people who think strongly about their health.”* (Male 18–19 years, FGD)

They suggested that peer educators and nurses visiting schools and communities, teachers, and a range of media (including comics, pamphlets, posters and radio) could be used to increase awareness*.* Providers and policymakers agreed that adolescents needed to be better informed about SRH and services:

*“If you don’t make young people aware that this service is available then they won’t know where to go and access. It needs to be made clear on maybe radio programs, outreach in communities – this needs to be clearly said and well advertised.”* (Policymaker, interview)

Many adolescents reported that 'encouragement’ and support from friends, parents and the community would make it easier to access services. This included parents or community leaders who allowed access to SRH information and services, as well as parents or experienced friends who provided advice or were able to accompany them to clinics. Boys and girls discussed the importance of parental support, particularly for improving girls’ access:

*“If parents let you, teachers, friends support you, parents want you to have a healthy life. [It is easier if] parents take her [fictional character], friends tell her what will happen at the check-up, or her boyfriend takes her.”* (Female 15–17 years, FGD)

#### ***Features of a youth-friendly health service***

The most important feature of a youth-friendly health service defined by adolescents was a friendly service provider (Table 3). Adolescents, providers and policymakers described a 'friendly’ provider as someone who was non-judgmental and kind, who understood adolescents and their rights, who kept confidentiality, who gave adolescents adequate time, and who was trained in SRH and counselling:

*“Must have nurses that specialise in sexual and reproductive health who can give treatment. Nurses must understand young people’s lives today. They shouldn’t talk crossly to young people because it is their job to help them. A health worker should be kind and friendly to young people.”* (Female 15–17 years, FGD)

*“If they [nurses] are helpful, keep everything confidential and secret, treat you well and talk kindly to you, give true and correct information.”* (Female 18–19 years, FGD)

*“We as staff we have to be friendly, not chase them away, welcome them, tell them that this is for them. It’s about how you approach them, you put them down to that youth level. We try even with the receptionist, we tell him you have to welcome everyone who comes in. You are the first one, if you are not smiling then inside their mind they don’t feel good.”* (Nurse, interview)

**Table 3:** Features of a youth-friendly health service ranked by adolescents from most to least important

**Overall**	**Females**	**Males**
Friendly health service providers	No cost	Friendly health service providers
Reliable commodity supply	Friendly health service providers	Reliable commodity supply
No cost	Male and female providers available	Convenient opening hours
Confidentiality	Confidentiality	No cost
Male and female providers available	Reliable commodity supply	Confidentiality
Convenient opening hours	Convenient opening hours	Male and female providers available
Things to do in the waiting room	Privacy	Things to do in the waiting room
Privacy	Things to do in the waiting room	Privacy
Standalone youth clinic	Standalone youth clinic	Standalone youth clinic

Providers and policymakers agreed that providers need training to work with adolescents. Most noted the lack of adolescent SRH education included in pre-service training curricula and limited opportunities to attend in-service training. Specific training needs included updating SRH knowledge, confidentiality, sexual and reproductive rights of adolescents, and communication and counselling skills. Many key informants believed that these competencies should be included in basic pre-service training of all providers. Positive impacts were reported by those who had attended training:

*“Now we are understanding that people have their rights….Not like before, we have come out from that and have been through a lot of training and workshops and we have to change from the old way before…Myself I went to some workshops in Fiji. After the training we are so keen to welcome, receive them [young people] than before.”* (Nurse, interview)

Adolescent girls in particular described the need to have a provider of the same sex, and some boys also had a preference for seeing a male nurse. Providers and policymakers identified that staff shortages, particularly in rural areas, made this challenging.

Having a reliable commodity supply was the second most important feature identified by adolescents, and the most important feature identified by rural groups:

*“Must have medicine every time, otherwise you spent money on transport for nothing.”* (Male 18–19 years, FGD)

Free services and commodities was the most important feature ranked by adolescent girls, and the fourth most important for boys. Almost all groups agreed that services and commodities (including condoms and contraceptives) should be free for adolescents since they were important for health and wellbeing and many would not be able to afford the fees:

*“Because young people don’t have money and if they have to pay [for contraception] then teen pregnancy will rise.”* (Male 15–17 years, FGD)

However, some adolescents explained that some financial contribution was important, particularly for commodities, either because it would encourage them to value the service or in recognition of the limited resources of clinics:

*“It’s good to pay for condoms because when you pay you take it seriously.”* (Female 18–19 years, FGD)

*“Sometimes you should pay so that they [clinics] can get more medicine.”* (Male 15–17 years, FGD)

Providers and policymakers were divided about whether SRH services should be free for adolescents. Many acknowledged the financial barriers facing adolescents but also recognised financial constraints affecting health services. Some reported that a subsidised fee was appropriate and that if adolescents paid for commodities, such as contraceptives, they were more likely to use them.

Having a standalone or youth-only clinic was the least important feature. Some adolescents reported that having a separate youth clinic would improve access to services by overcoming concerns about privacy at mainstream health facilities. However, many reported that other features were more important and that a lack of privacy could be overcome by providing separate entrances and waiting areas for adolescents (where youth-oriented activities and resources could be provided) or having separate youth-only clinic hours. Many providers and policymakers believed that standalone youth clinics were the ideal, but noted that a lack of financial and human resources meant that this was not feasible in all communities. Some agreed that there was scope to make existing services more accessible:

*“I think young people in the clinic they should need another room just for themselves. When they come with mothers and everybody they are frightened to come with their problems. In another clinic we used a back door instead of the main entrance. I think the set up should be different to general clinics.”* (Nurse, interview)

## Discussion

Despite two decades of international research describing the barriers adolescents face accessing care, and global guidance on how these might be overcome, adolescents’ access to SRH services remains poor in many settings [16,17,23]. The need for context-specific research to support effective implementation of youth-friendly health services has been noted [24,25]. This study has identified key demand and supply-side barriers facing adolescents in Vanuatu and highlighted opportunities to increase accessibility and acceptability of SRH services.

Similar to studies from other regions [25-30], the real or perceived lack of confidentiality and judgmental attitudes of service providers were strong disincentives to seek care. Skills and attitudes of providers were also the defining feature of what adolescents in Vanuatu considered to be a youth-friendly service and would therefore be an important target for intervention. Providers and policymakers highlighted the lack of adolescent SRH competencies included in current nursing curricula and limited in-service training opportunities; lack of training has been associated with negative attitudes towards adolescent SRH [30,31]. Training that addresses knowledge, attitudes and communication and counselling skills (including confidentiality) can improve provider performance, with continuing education more likely to have a positive impact on attitudes [23,31]. While ongoing in-service training is important, inclusion of adolescent health competencies in basic pre-service training of all primary-level providers is essential to ensure that those who may come into contact with adolescents have the skills to deal with them effectively and sensitively [32].

In keeping with international literature [23], financial barriers impacted significantly on adolescents’ access to services in Vanuatu. In addition to policies and procedures that ensure services are free or affordable, innovative financing mechanisms, such as voucher schemes, may increase demand and uptake of SRH services by adolescents [33]. Promising findings from studies in Latin America have suggested that combining such interventions with provider incentives can increase access and improve quality of care [34,35].

A youth-only service, while recognised as the 'gold standard’ of youth-friendly care by providers and policymakers, was less important to adolescents than other 'youth friendly’ features. This is encouraging given the financial, human resources, and logistical challenges of providing standalone youth clinics in resource-limited settings, particularly those with small, geographically dispersed populations. Consideration of the physical environment to ensure privacy, convenient opening hours and well-trained staff are opportunities to make existing services more acceptable to adolescents. These features were also identified in a 2006 Ministry of Health report that explored service delivery preferences of urban adolescents in Port Vila [14]. Interestingly, reliable commodity supply was a high priority for adolescents, more so than other features such as privacy. Commodity insecurity is a well-noted challenge in small island Pacific states [36]. It is perhaps unsurprising that stock-outs are a disincentive for adolescents to access services, and highlights the importance of ensuring quality care in addition to 'friendly’ care.

Providing youth-friendly health services alone may not be sufficient to increase adolescent’s use of services [37]. Socio-cultural norms and taboos were the most significant barriers to accessing care noted by adolescents in Vanuatu. This is consistent with a 1998 knowledge, attitudes and practices survey that identified fear and discomfort as the major reasons for non-use of family planning among adolescents [38]. The influence of *kastom* on accepted norms of sexual behaviour, gender and open discussion of sexual matters in Melanesia, particularly related to HIV, has been described in detail by other authors [39-41].

Community support is an important predictor of adolescents’ care-seeking behaviour, and there is some evidence that youth-friendly health services are more effective if linked with community interventions [33,42,43]. In addition to increasing adolescents’ knowledge and demand for SRH services, there is also a need to engage with communities, particularly gate-keepers such as parents and community leaders. Use of multimedia to generate community support and stimulate parent-adolescent communication, community education and mobilisation, and increasing the participation of communities and adolescents in the design and implementation of interventions may assist in creating a supportive environment for adolescent SRH [33]. There is however a great need for evaluations of interventions in the Pacific to identify effective approaches in this region.

This study has some important limitations. Participants were recruited from the two most populous islands, excluding adolescents from more rural and remote islands where access to services is particularly limited and *kastom* more prominent. For this reason our findings may not be applicable to other parts of Vanuatu, particularly more remote islands. While we aimed to sample a broad cross-section of adolescents, convenience sampling by peer educators through schools and youth centres may have excluded more marginalised adolescents and those less likely to be engaged with health promotion programs and SRH services. The inclusion of schools that had had previous engagement with health promotion programs related to SRH may also have biased our sample, over sampling adolescents who were more likely to be engaged with SRH service providers (particularly those provided by non-government organisations). We attempted to address these biases by also recruiting in and out-of-school adolescents from urban and rural communities that had not previously been targeted by the local research partner.

The limited ability to analyse data during the data collection process also restricted opportunities to adapt the question guide and the potential to explore new information. An additional limitation of this study was the reliance on written notes during the FGDs rather than audio-recording. This contributed to brevity of transcripts, with note-takers recording only key points in some cases rather than capturing phrases and the language of the discussion. This approach may also have introduced biases as note-takers may have been more likely to record comments that were perceived to be of most relevance to the study or of most interest to the note-taker, rather than an accurate record of the discussion.

Traditional healers were identified by many adolescents as service providers, however an in-depth exploration of their role in adolescent SRH was beyond the scope of this study and would be an important focus of future research. Finally, including parents and community leaders would have provided useful information to identify potential targets for intervention.

## Conclusions

Adolescents in Vanuatu face considerable barriers to accessing SRH services, with socio-cultural factors and the lack of sensitive, skilled providers among the major deterrents to seeking care. However, our findings suggest that much could be done to make existing health services in Vanuatu more youth-friendly, providing a base for the development of national policy and guidelines. Further research is needed however to determine the impact, sustainability and cost-effectiveness of youth-friendly health services in this region. Importantly, our study has highlighted that providing youth-friendly health services alone will not alleviate the burden of poor SRH among adolescents in Vanuatu. Investment is required in well-evaluated, context-specific strategies that aim to create a more supportive environment for adolescents and their sexual and reproductive health.

## Abbreviations

FGD: Focus group discussion; FP: Family planning; SRH: Sexual and reproductive health; STI: Sexually transmitted infection.

## Competing interests

Wan Smolbag Theatre is a non-government organisation providing youth-friendly health services in Vanuatu. The other authors declare that they have no competing interests.

## Authors’ contributions

EK and NG designed the study. SB, JM and DH provided support to the development of the study tools and participant recruitment strategy and coordinated data collection. JH translated the transcripts. EK, NG and DH analysed the transcripts. All authors contributed to interpretation of the findings. EK wrote the manuscript that was reviewed and approved for publication by all authors.

## Pre-publication history

The pre-publication history for this paper can be accessed here:

http://www.biomedcentral.com/1472-6963/13/455/prepub
